# Genome editing of human pluripotent stem cells to generate human cellular disease models

**DOI:** 10.1242/dmm.012054

**Published:** 2013-06-10

**Authors:** Kiran Musunuru

**Affiliations:** 1 Department of Stem Cell and Regenerative Biology, Harvard University, Cambridge, MA 02138, USA; 2 Division of Cardiovascular Medicine, Brigham and Women’s Hospital, Boston, MA 02115, USA, e-mail: kiranmusunuru@gmail.com

## Abstract

Disease modeling with human pluripotent stem cells has come into the public spotlight with the awarding of the Nobel Prize in Physiology or Medicine for 2012 to Drs John Gurdon and Shinya Yamanaka for the discovery that mature cells can be reprogrammed to become pluripotent. This discovery has opened the door for the generation of pluripotent stem cells from individuals with disease and the differentiation of these cells into somatic cell types for the study of disease pathophysiology. The emergence of genome-editing technology over the past few years has made it feasible to generate and investigate human cellular disease models with even greater speed and efficiency. Here, recent technological advances in genome editing, and its utility in human biology and disease studies, are reviewed.

## Disease modeling with human pluripotent stem cells

There are two main varieties of human pluripotent stem cells (hPSCs): human embryonic stem cells (hESCs), which are derived directly from embryos (Thomson et al., 1998; Reubinoff et al., 2000) and continue to be considered the gold-standard hPSCs, and induced pluripotent stem cells (iPSCs), which are generated by the introduction of ‘reprogramming factors’ into fibroblasts or other differentiated somatic cell types (Takahashi et al., 2007; Yu et al., 2007; Park et al., 2008a; Nakagawa et al., 2008). A third type, stem cells derived by somatic cell nuclear transfer (SCNT) – the transfer of a nucleus from a differentiated cell into a denucleated ovum – have recently been successfully generated for humans (Tachibana et al., 2013).

All hPSCs share two useful theoretical properties. First, they can be maintained in culture for a large number of passages without loss of genomic integrity, which distinguishes them from standard cultured cell lines that are transformed or immortalized and have severely abnormal karyotypes. [In reality, upon continued passaging, both hESCs and iPSCs eventually accumulate genetic alterations that confer a growth advantage in culture (Draper et al., 2004; Cowan et al., 2004; Mitalipova et al., 2005; Maitra et al., 2005; Mayshar et al., 2010; Laurent et al., 2011; Taapken et al., 2011; Martins-Taylor et al., 2011; Amps et al., 2011).] Second, hPSCs can be differentiated into any of the myriad of somatic cell types in the human body. [In practice, the ability to differentiate into a desired cell type depends on the availability of an efficient protocol to achieve the differentiation, which at present is only true of a small number of cell types (e.g. Lee et al., 2010; Lian et al., 2013) but will surely expand to cover more in the coming years.] This feature is advantageous because it makes it possible to derive cell types for which standard cultured cell lines do not exist and which are difficult to obtain from patients as primary cells (e.g. neurons).

Owing to recent advances, iPSCs can now be derived from a skin biopsy (Dimos et al., 2008; Park et al., 2008b) or blood sample (Seki et al., 2010; Loh et al., 2010; Staerk et al., 2010) from virtually any given patient, making it possible to derive, expand and differentiate somatic cells that are genetically matched to the patient. In principle, this provides a means by which an investigator can extensively study a patient’s pathophysiology without having to touch the patient after the iPSCs are generated.

However, there are several limitations to the utility of iPSC-based studies. First, the disease under study must have a strong genetic component. In the best-case scenario, the disease is monogenic in nature and driven by a single gene mutation (e.g. cystic fibrosis), which would be retained in patient-derived iPSCs and cause disease-related phenotypes to manifest at the cellular level in the appropriate differentiated cell type (e.g. lung epithelial cells). In contrast, for a disease that is driven by numerous genetic and environmental factors (e.g. myocardial infarction), the extent to which studies using patient-derived iPSCs will offer any advantage in understanding the disease process is unclear.

**Fig. 1.**
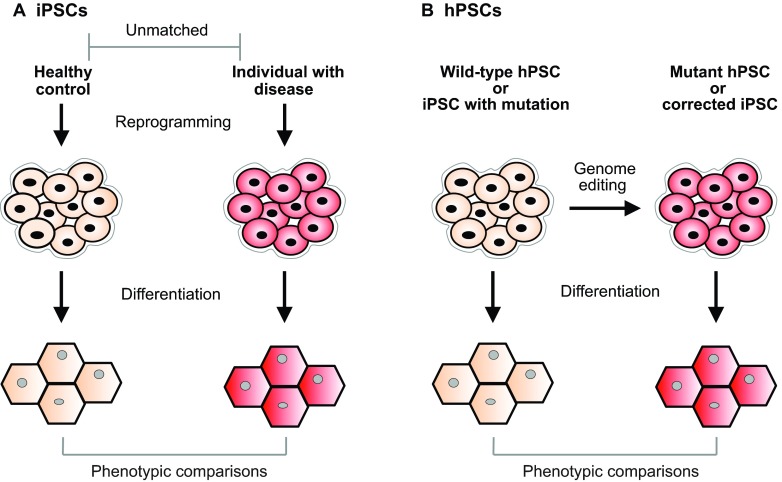
**A comparison of two study designs for disease modeling using human pluripotent stem cells.** (A) Induced pluripotent stem cells (iPSCs) are reprogrammed from an individual(s) with disease and a control individual(s). The iPSCs are differentiated into a cell type of interest; the cell lines are compared for relevant phenotypes. This study design is susceptible to a number of potential confounders emanating from the fact that the cell lines are not matched (genetically, epigenetically, etc.) and could have been derived by different methods and in different circumstances. The study design is also time-consuming and costly. (B) Human pluripotent stem cells (hPSCs) – whether human embryonic stem cell lines (hESCs) or pre-existing iPSCs – are modified with genome editing, thereby creating optimally matched cell lines. The wild-type and mutant hPSCs are differentiated into a cell type of interest; the cell lines are compared for relevant phenotypes. This study design minimizes confounders – making it more scientifically rigorous – as well as reducing the associated time and costs.

Second, as with any scientific study, the quality of iPSC-based studies depends on the availability of appropriate controls – any phenotypes observed in a patient’s iPSC-derived cells should only be interpreted via comparison with control cells (Fig. 1). There are a number of published studies in which one or a few iPSC lines from patients with a disease and one or a few iPSC lines from individuals without the disease have been generated and differentiated, with claims that phenotypic differences observed between the cell lines are relevant to disease (e.g. Ebert et al., 2009; Lee et al., 2009; Ye et al., 2009; Carvajal-Vergara et al., 2010; Rashid et al., 2010; Moretti et al., 2010; Swistowski et al., 2010; Marchetto et al., 2010; Brennand et al., 2011; Sun et al., 2012; HD iPSC Consortium, 2012). However, these studies are potentially flawed because they do not account for possible confounders that might be responsible for the phenotypic differences.

Differences in genetic background are of greatest concern; even in studies in which healthy siblings have been used as controls for disease patients, only ∼50% of the genome is shared between any siblings, and phenotypic differences could be the result of DNA variants in the other ∼50% of the genome, rather than the disease-associated mutations. Furthermore, a number of studies have documented that the process of generating, expanding and passaging iPSC lines can lead to the accumulation of a variety of genetic alterations, ranging from single-nucleotide variants to copy-number variants to chromosomal amplifications, deletions and rearrangements (Mayshar et al., 2010; Laurent et al., 2011; Hussein et al., 2011; Gore et al., 2011; Taapken et al., 2011; Howden et al., 2011; Martins-Taylor et al., 2011; Yusa et al., 2011; Amps et al., 2011; Ji et al., 2012). Another significant confounder is epigenetic state. A number of studies have documented that iPSCs vary widely with respect to genomic methylation patterns, in some cases seeming to retain epigenetic ‘memory’ reflecting the somatic cell of origin from which the iPSCs were reprogrammed; some iPSCs seem to retain this memory indefinitely, whereas others gradually lose this memory as they go through many passages in culture (Kim et al., 2010; Polo et al., 2010; Bock et al., 2011; Lister et al., 2011; Nishino et al., 2011; Bar-Nur et al., 2011; Kim et al., 2011; Nazor et al., 2012; Ruiz et al., 2012). Some of these studies suggest that epigenetic state can affect the differentiation potential of an iPSC line, i.e. differentiation into some cell types over others is favored. Other potential confounders include: unmatched age, gender and ethnicity between the patients and control individuals; differences in methodology used to induce pluripotency (e.g. lentivirus versus RNA transfection); and differences in passage number and adaptation to culture of the iPSC lines.

The most rigorous possible comparisons would be between cell lines that differ only with respect to disease mutations, i.e. otherwise isogenic cell lines. One way to ensure this would be to use wild-type and mutant cell lines derived from the same parental cell line (Fig. 1). This strategy would also eliminate, or at least mitigate, all of the other confounders, allowing investigators to directly connect genotype to phenotype to establish causality. Such a strategy would require the ability to efficiently introduce specific genetic alterations into the genomes of hPSCs at will. Fortunately, the emerging technology known as ‘genome editing’ is now putting this ability within easy reach.

An elegant demonstration of the superiority of an isogenic cell line study design over a study design comparing iPSC lines from patients versus control individuals was provided by a recent study of a mutation in the *LRRK2* gene, which is implicated in Parkinson disease (Reinhardt et al., 2013). The investigators derived iPSC lines from patients with the *LRRK2* mutation and healthy individuals without the mutation; they also used genome editing to correct the mutation in the mutant iPSC lines. They then differentiated the cell lines into neurons and compared global gene expression profiles, followed by cluster analysis to assess the degree of similarity among the cell lines. Notably, they found that the healthy iPSC lines and the mutant iPSC lines did not cluster in separate groups, as would be hoped; rather, one of the healthy lines clustered very closely with one particular mutant line, whereas the other healthy line was very different from all of the other lines. Two mutant lines from the same patient were quite different, with one of these mutant lines being more similar to one of the healthy lines and to a mutant line from a different patient. The only cell lines that reliably clustered close together (i.e. were extremely similar) were pairs of mutant lines with and without correction of the mutation by genome editing (Reinhardt et al., 2013).

## The emerging promise of genome editing

Classical gene-targeting technology uses homologous recombination to target an investigator-specified gene for disruption or modification (Smithies et al., 1985; Thomas and Capecchi, 1987). This approach has proven to be invaluable through its use in mouse embryonic stem cells to generate germline knockout or knock-in mice. However, homologous recombination is challenging in hPSCs (Zwaka and Thomson, 2003) and, although homologous recombination has become a mainstay for investigating gene function, its use in mammals has been limited primarily to studies in mice. Knockout strategies utilizing homologous recombination in human somatic cells are similarly challenging and, as a result, technologies such as antisense oligonucleotides and short interfering RNAs (nucleic acids that match to sequences in cellular RNA transcripts and result in their degradation) have flourished as a means to knock down gene expression. However, these reagents interfere with gene expression only transiently, and the knockdown effect can be incomplete or extend to off-target genes (Qiu et al., 2005). In light of recent advances in the use of hPSCs for disease modeling, as described in the previous section, the demand for more efficient and rapid methods of gene knockout or modification has only increased.

The emerging technology of genome editing, also known as genome engineering, seeks to meet this need by providing the ability to more efficiently introduce a variety of genetic alterations, ranging from single-nucleotide modifications to whole gene addition or deletion, all with a high degree of target specificity. The key features of the most widely used genome-editing systems, in addition to the major advantages and disadvantages of each (Table 1), are described below.

**Table 1.**
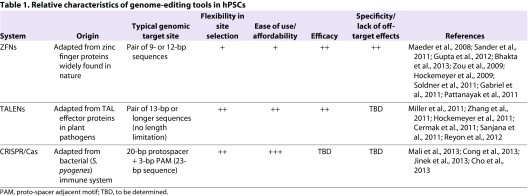
Relative characteristics of genome-editing tools in hPSCs

### Zinc finger nucleases

Zinc finger nucleases (ZFNs) are a type of genome-editing technology that is increasingly being used in academic and industry research (Urnov et al., 2010). ZFNs are fusion proteins consisting of an array of site-specific DNA-binding domains – adapted from zinc finger transcription factors – fused to the nuclease domain of the bacterial *Fok*I restriction enzyme. Each zinc finger domain recognizes a 3- to 4-base-pair (bp) DNA sequence, and individual domains can be arranged in tandem to bind to an extended nucleotide sequence that is unique within a genome.

**Fig. 2.**
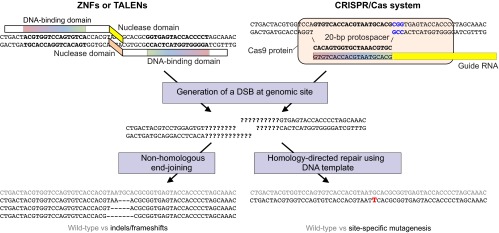
**Genome editing to knock out genes or knock in DNA variants.** Engineered nucleases – whether ZFNs or TALENs – are designed to bind to a specific DNA sequence in the genome, typically as a dimer, as depicted at the top left. The DNA-binding domains of the proteins bind to flanking DNA sequences (indicated in bold) and position their nuclease domains such that they dimerize and generate a double-strand break (DSB) between the binding sites. In CRISPR/Cas systems, as depicted at the top right, the guide RNA recognizes and hybridizes a 20-bp protospacer in the genome (indicated in bold); the Cas9 protein binds the guide RNA, unwinds the DNA, binds to the NGG motif (indicated in blue) and generates the DSB. The consequence of the DSB is variable with respect to the sequence around the break because native enzymes might further process the free DNA ends. The DSB can be repaired by non-homologous end-joining (NHEJ), which usually restores the original sequence (indicated in gray) but occasionally introduces an insertion or deletion (indel) that can cause a frameshift knockout in the coding sequence of a gene. Alternatively, the DSB can be repaired by homology-directed repair (HDR) using a homologous template – either the endogenous sister chromosome or an exogenously introduced DNA repair template, whether a double-stranded vector or a single-stranded DNA oligonucleotide. If the repair template contains a mutation, the mutation (indicated in red) can be stably incorporated into the genome, resulting in site-specific mutagenesis.

To cleave a target site of interest, ZFNs are typically designed in pairs that recognize sequences flanking the site; upon binding of the ZFN pair around the site, the *Fok*I nuclease domains dimerize and generate a double-strand break (DSB) (Urnov et al., 2010). DSBs are repaired by the cell using either the error-prone process of nonhomologous end-joining (NHEJ) or homology-directed repair (HDR) with the corresponding locus on the sister chromosome serving as a repair template (Fig. 2). NHEJ can be used to introduce frameshifts into the coding sequence of a gene, thereby generating premature truncations that effectively knock out the gene. HDR can be exploited by the introduction of an exogenous repair template that harbors a desired mutation flanked by homology arms, thereby greatly improving upon the efficiency of traditional homologous recombination, in which the initiation of the process (generation of a DSB) must occur spontaneously. The exogenous repair template can either be in the form of a double-strand DNA vector or a single-stranded DNA oligonucleotide. In the case of the latter, homology arms of as little as 20-nucleotides in length are sufficient for the introduction of DNA sequences into the genomes of hPSCs (Soldner et al., 2011). The efficiency seems to be high enough that antibiotic selection to expedite the screening for correctly targeted clones might be unnecessary in some cases, with no subsequent need to remove an antibiotic cassette from the genome using the Cre-*lox* or FLP-*FRT* system (which typically leaves a ‘scar’ behind in the genome), saving a considerable amount of time.

Despite the advantages offered by ZFN technology, ZFNs have proven difficult for non-specialist investigators to engineer from scratch because it has not been straightforward to successfully assemble zinc finger domains to bind an extended stretch of nucleotides (Ramirez et al., 2008). Although a library of zinc finger components and protocols to perform screens to identify optimized ZFNs has been made freely available to the academic community (Maeder et al., 2008; Maeder et al., 2009), it can take months for non-specialists to engineer ZFNs that target a genomic site with high efficiency. Furthermore, target-site selection is limited – these freely available ZFN components can only be used for binding sites every few hundred bp throughout the genome. Alternative platforms to construct ZFNs have since emerged, and these show variation in speed, site selection and success in generating efficacious ZFNs (Sander et al., 2011; Gupta et al., 2012; Bhakta et al., 2013). A commercial option to obtain optimized ZFNs for a specified target site evidently has a high success rate but remains expensive.

### Transcription activator-like effector nucleases (TALENs)

Recent studies of a class of proteins called transcription activator-like effectors (TALEs) have characterized a newly identified DNA-binding module, termed a TAL repeat, that is used by each protein in a tandem array with 10–30 repeats to recognize extended DNA sequences with a 1-repeat to 1-bp correspondence (Bogdanove and Voytas, 2011). Each repeat has 33–35 amino acids, with two adjacent amino acids [termed the repeat-variable di-residue (RVD)] conferring specificity for one of the four DNA bases (Moscou and Bogdanove, 2009; Boch et al., 2009). Deciphering of the RVD ‘code’ has led to the creation of a new class of engineered site-specific nucleases comprising an array of TAL repeats fused to the *Fok*I nuclease domain, termed TAL effector nucleases (TALENs) (Christian et al., 2010; Miller et al., 2011). TALENs function in a similar way to ZFNs in that they generate DSBs within a target site, and so they can also be used to knock out genes or knock in mutations (Fig. 2). However, TALENs seem to be far easier to design than ZFNs: the RVD ‘code’ has been successfully used to create many *de novo* extended TAL repeat arrays that bind with high affinity to desired genomic DNA sequences (Miller et al., 2011; Zhang et al., 2011; Hockemeyer et al., 2011). Moreover, there seem to be fewer constraints with respect to which sites can be targeted in human cells, with at least a few potential sites available within each 100 bp of genomic DNA, although methylation of the DNA target site can attenuate the binding affinity (Bultmann et al., 2012). TALENs can in principle be designed and constructed in as short a time as a few days and in as large a number as hundreds at a time (Cermak et al., 2011; Sanjana et al., 2011; Reyon et al., 2012), and they have been demonstrated to have robust gene-targeting efficacy in hPSCs (Hockemeyer et al., 2011).

### CRISPR/Cas systems

Even more recently, genome editing tools have been adapted from bacterial adaptive immune systems known as clustered regularly interspaced short palindromic repeats (CRISPRs) and CRISPR-associated (Cas) systems, which use a combination of proteins and short guide RNAs to recognize and cleave complementary DNA sequences. The bacteria accumulate ‘protospacers’ that correspond to foreign DNA sequences, such as plasmids and phage genomes, which are then targeted for destruction. By early 2013, four groups had shown that heterologous expression of the *Streptococcus pyogenes* Cas9 protein along with a guide RNA complex (comprising either a single chimeric RNA or two separate RNAs) in mammalian cells results in DSBs at a site with (1) a 20-bp sequence matching the protospacer of the guide RNA and (2) an adjacent NGG amino acid sequence [termed the protospacer-adjacent motif (PAM)] recognized by Cas9 (Fig. 2) (Cong et al., 2013; Mali et al., 2013; Jinek et al., 2013; Cho et al., 2013).

Thus, in principle, a CRISPR/Cas system can be easily adapted to target a genomic sequence by simply changing the guide RNA, which entails switching out only a 20-bp sequence, with the Cas9 protein component unchanged. This makes CRISPRs easier to engineer than ZFNs or even TALENs, particularly if one desires to generate a library of vectors to target numerous sites in the genome. Another potential advantage of a CRISPR/Cas system is that a single vector can accommodate multiple guide RNAs in series, which are then processed into individual RNAs that allow for simultaneous, multiplexed targeting of multiple sites in the same cell (Cong et al., 2013). At present, even with the most versatile CRISPR/Cas system, genomic site selection is limited to 23-bp sequences on either strand that end in an NGG motif (the PAM for *S. pyogenes* Cas9), which occur on average once every 8 bp (Cong et al., 2013). Studies to determine the relative efficacies, specificities and ease of use of ZFNs, TALENs and CRISPRs in hPSCs are ongoing (Table 1) and will no doubt influence the relative popularity of the genome-editing tools among the biomedical community.

### Additional genome-editing tools

Other tools that have successfully been used for genome editing in human cells include meganucleases (Grizot et al., 2009), adeno-associated viruses (Russell and Hirata, 1998; Khan et al., 2010) and adenoviruses (Suzuki et al., 2008; Liu et al., 2011; Liu et al., 2012). Although each of these tools carries its own advantages, at the present time none of them offers the same adaptability and ease of use as ZFNs, TALENs and CRISPRs. Nonetheless, given the breathtaking rate of progress in the field, it would not be surprising if an even more attractive genome-editing tool were to emerge in the near future.

## Differentiation and phenotyping of cell models

To date, there have been a number of reports demonstrating the feasibility of performing genome editing in hPSCs with ZFNs, TALENs, CRISPRs and other tools, although these studies were largely performed as proof-of-principle exercises (e.g. Lombardo et al., 2007; Suzuki et al., 2008; Zou et al., 2009; Hockemeyer et al., 2009; Hockemeyer et al., 2011; Soldner et al., 2011; Yusa et al., 2011; Zou et al., 2011; Sebastiano et al., 2011; Li et al., 2011; Mali et al., 2013). As discussed below, in only a few cases have genome-editing tools been used to generate isogenic wild-type versus mutant cell lines that have then been differentiated into disease-relevant cell types and shown to display phenotypic differences that give insight into disease pathophysiology.

In landmark studies, iPSC lines from patients with monogenic disorders have been ‘cured’ via genome-editing-based correction of the causal mutation and then compared with the parental lines. Fibroblasts obtained from patients with Hutchinson-Gilford progeria syndrome (HGPS) and atypical Werner syndrome (AWS), each caused by mutations in the *LMNA* gene, were used to generate iPSCs (Liu et al., 2011). The investigators used an adenoviral vector containing the wild-type *LMNA* sequence to correct the mutation in each of the cell lines. The original and corrected HGPS cell lines were differentiated into vascular smooth muscle cells and fibroblasts; large proportions of the original differentiated cells displayed dysmorphic nuclei and senescence that are characteristic of HGPS, in contrast to corrected cells. An iPSC line from a Huntington disease (HD) patient with an allele bearing 72 polyglutamine repeats was altered to carry only 21 polyglutamine repeats (An et al., 2012). Upon differentiation into neural stem cells, the original HD cells displayed a significant increase in caspase-3/7 activity upon growth factor deprivation and were significantly more susceptible to cell death compared with corrected cells; in addition, the former displayed reduced mitochondrial bioenergetics compared with the latter, consistent with established HD pathophysiology.

Subsequent studies have gone a step further by both introducing disease mutations into wild-type cell lines and, in parallel, correcting the same mutations in patient-derived iPSC lines. Two studies have focused on the G2019S mutation of the *LRRK2* gene, which is associated with familial and sporadic Parkinson’s disease (PD). In one study (Liu et al., 2012), the investigators generated iPSC lines from patients with the mutation, used adenovirus to correct the mutation in one of the lines, and then differentiated the matched cell lines into neural stem cells (NSCs). The PD NSCs displayed progressive nuclear aberrations after being maintained for more than a dozen passages in culture; these aberrations were absent in the corrected NSCs. Furthermore, when the PD NSCs were propagated for more than a dozen passages, they became impaired in their ability to differentiate into neurons, compared with similarly passaged corrected NSCs. When the investigators used adenovirus to insert the *LRRK2* G2019S mutation into wild-type hESCs, they found that NSCs from the mutant hESCs – but not NSCs from the wild-type hESCs – displayed the same nuclear and differentiation defects as the PD iPSCs, thereby establishing the necessity and sufficiency of the G2019S mutation for the disease phenotypes. The *coup de grace* was the investigators’ demonstration of aberrant nuclear morphology in neurons in the hippocampal dentate gyrus of post-mortem human brain samples from individuals with PD, a pathological feature not previously described in PD. Thus, this study stands as the first example of a disease phenotype being initially discovered in an hPSC-based model system and then subsequently confirmed in patients.

In the second study on the G2019S mutation of the *LRRK2* gene (Reinhardt et al., 2013), the investigators generated iPSC lines from PD patients with the mutation and from control individuals, and used ZFNs to correct the mutation in three of the patient-derived lines and to insert the mutation into a control iPSC line. The matched cell lines were then differentiated into midbrain dopaminergic (mDA) neurons. Interestingly, the investigators assessed a rather different set of phenotypes than those studied by Liu et al. (Liu et al., 2012), despite focusing on the same mutation. They found that mutant neurons consistently displayed reduced neurite outgrowth, as well as increased apoptosis in response to oxidative stress, when compared with isogenic wild-type neurons. Expression profiling of pairs of isogenic wild-type and mutant cell lines revealed several genes that are consistently dysregulated by the mutant *LRRK2* gene, including *CPNE8*, *CADPS2*, *MAP7* and *UHRF2*; remarkably, individual knockdown of each of those genes in mutant neurons modulated their sensitivity to oxidative stress. The investigators also established that the increased sensitivity to stress of the mutant neurons was at least in part due to activation of ERK signaling and could be reversed with an inhibitor of ERK phosphorylation, pointing to a potential new therapeutic approach for individuals with PD.

## Shortcomings of genome-edited cell models

A strong rationale for using genome-edited cell models for phenotypic studies is that, by assessing the effect of a DNA variant on an isogenic background, potential confounders will be minimized. However, this presupposes that genome-editing tools will yield cell lines that are truly isogenic. One large concern about the use of ZFNs, TALENs and CRISPRs – all of which are designed, after all, to introduce DSBs into genomic DNA – is that they will not only cleave at ‘on-target’ sites but also at ‘off-target’ sites. Thus, there is the possibility that the tools will introduce significant genomic alterations besides the desired DNA variants, rendering the resulting cell lines not truly isogenic and introducing a source of confounding.

Data remain scarce as to the extent of off-target effects of ZFNs, TALENs and CRIPSRs. In one study in which ZFN targeting was performed in hPSCs, the investigators searched ten predicted possible off-target genomic sites (based on sequence similarity to the on-target site) for evidence of mutagenesis and identified one event in 184 clones assessed (Hockemeyer et al., 2009). Although this might seem to be a low rate, when extrapolated to the entire diploid genome of 6 billion bp, this result implies that there is a concrete risk of an off-target event in any given targeted clone. Two studies of ZFNs that used unbiased methods to identify off-target genomic sites for several ZFN pairs documented infrequent off-target effects at numerous loci in a cultured human tumor cell line (Gabriel et al., 2011; Pattanayak et al., 2011). A study in which TALENs were introduced into a pool of hPSCs documented low but measurable rates of mutagenesis at some of 19 predicted possible off-target sites (based on sequence similarity to the on-target site) (Hockemeyer et al., 2011). Preliminary results in mammalian cells demonstrate that CRISPR/Cas systems can tolerate single-nucleotide mismatches from the expected target sequence (Cong et al., 2013).

Thus, off-target effects produced by genome-editing tools, however infrequent, might make it unrealistic to expect to obtain 100% isogenic wild-type and targeted cell lines in any given experiment. This does not undermine the genome-editing study design; rather, it establishes the degree of rigor that will be needed to be sure that any phenotypic differences observed between wild-type clones and targeted clones are truly related to the DNA variant of interest. Any one-by-one comparison of a wild-type clone and a targeted clone could be confounded by an off-target effect in one of the clones. However, given the low frequency of off-target effects at any given locus, it is unlikely that multiple clones will have the same off-target effect, and so a study in which multiple wild-type clones show consistent phenotypic differences from multiple targeted clones would argue for those differences being due to the DNA variant of interest. Thus, a prudent study design would entail generating and comparing at least two or three of each type of clone to mitigate any concern of genetic heterogeneity – whether off-target effects or other genetic alterations accumulated during passaging of the cells – being a confounder, regardless of which particular genome-editing tool is employed.

## Challenges and outlook

It seems likely that, within a few years, the use of genome editing to generate human cell-based disease models will become a standard, routine approach in the laboratory that could rival the use of genetically modified mice in popularity. Indeed, the former has the advantage of being far more rapid than the latter – involving a timeframe of months instead of years – as well as potentially being better at reflecting human physiology. However, it also carries the disadvantage of being limited to the study of cell-autonomous phenotypes, which will be inadequate for assessing complex physiological conditions. One means of moving beyond ‘cells-in-a-dish’ studies would entail the incorporation of multiple hPSC-derived differentiated cell types into a single model (Di Giorgio et al., 2008). Another strategy would be to incorporate hPSC-derived cells into chimeric animal models, e.g. replacing a mouse’s endogenous hepatocytes with engrafted hPSC-derived hepatocytes (Yusa et al., 2011), thus allowing for interrogation of the effects of human genetic variation in whole-animal models.

As it becomes easier to introduce mutations into hPSCs, it will become feasible to test the effects of the mutations in multiple cell lines with different genetic backgrounds. This will allow investigators to assess the importance of genetic modifiers on disease penetrance, i.e. if a mutation evokes a disease phenotype in some cell lines but not others. In some cases, it might be more informative to start with a patient-specific iPSC line and use genome editing to ‘cure’ a disease mutation. The most robust possible study design would be to do both: insert a disease mutation into a wild-type cell line – thereby testing for sufficiency of the mutation for disease – and correct the disease mutation in a patient-specific iPSC line – thereby testing for necessity of the mutation for disease (Liu et al., 2012; Reinhardt et al., 2013).

Finally, genome editing will make it possible to go beyond disease modeling and facilitate the discovery of therapeutics. For example, genome-editing tools should make it straightforward to insert reporters into genomic loci of interest, allowing for RNA-interference screens or small-molecule screens to identify genes and probes that have a desired functional effect. Genome-editing tools might themselves become the therapies, as is the case in the use of ZFNs to disrupt the *CCR5* gene in T cells, thereby rendering them resistant to human immunodeficiency virus (HIV) infection and useful for transplantation into HIV-positive individuals (Perez et al., 2008), a strategy that is now in clinical trials. Indeed, the ability to modify the human genome upon demand is so transformative that it certainly will be applied in ways that we can only begin to imagine.

## References

[b1-0060896] Amps K. Andrews P. W. Anyfantis G. Armstrong L. Avery S. Baharvand H. Baker J. Baker D. Munoz M. B. Beil S. International Stem Cell Initiative (2011). Screening ethnically diverse human embryonic stem cells identifies a chromosome 20 minimal amplicon conferring growth advantage. Nat. Biotechnol. 29, 1132–1144 22119741 10.1038/nbt.2051 PMC3454460

[b2-0060896] An M. C. Zhang N. Scott G. Montoro D. Wittkop T. Mooney S. Melov S. Ellerby L. M. (2012). Genetic correction of Huntington’s disease phenotypes in induced pluripotent stem cells. Cell Stem Cell 11, 253–263 22748967 10.1016/j.stem.2012.04.026 PMC3608272

[b3-0060896] Bar-Nur O. Russ H. A. Efrat S. Benvenisty N. (2011). Epigenetic memory and preferential lineage-specific differentiation in induced pluripotent stem cells derived from human pancreatic islet beta cells. Cell Stem Cell 9, 17–23 21726830 10.1016/j.stem.2011.06.007

[b4-0060896] Bhakta M. S. Henry I. M. Ousterout D. G. Das K. T. Lockwood S. H. Meckler J. F. Wallen M. C. Zykovich A. Yu Y. Leo H. (2013). Highly active zinc-finger nucleases by extended modular assembly. Genome Res. 23, 530–538 23222846 10.1101/gr.143693.112 PMC3589541

[b5-0060896] Boch J. Scholze H. Schornack S. Landgraf A. Hahn S. Kay S. Lahaye T. Nickstadt A. Bonas U. (2009). Breaking the code of DNA binding specificity of TAL-type III effectors. Science 326, 1509–1512 19933107 10.1126/science.1178811

[b6-0060896] Bock C. Kiskinis E. Verstappen G. Gu H. Boulting G. Smith Z. D. Ziller M. Croft G. F. Amoroso M. W. Oakley D. H. (2011). Reference Maps of human ES and iPS cell variation enable high-throughput characterization of pluripotent cell lines. Cell 144, 439–452 21295703 10.1016/j.cell.2010.12.032 PMC3063454

[b7-0060896] Bogdanove A. J. Voytas D. F. (2011). TAL effectors: customizable proteins for DNA targeting. Science 333, 1843–1846 21960622 10.1126/science.1204094

[b8-0060896] Brennand K. J. Simone A. Jou J. Gelboin-Burkhart C. Tran N. Sangar S. Li Y. Mu Y. Chen G. Yu D. (2011). Modelling schizophrenia using human induced pluripotent stem cells. Nature 473, 221–225 21490598 10.1038/nature09915 PMC3392969

[b9-0060896] Bultmann S. Morbitzer R. Schmidt C. S. Thanisch K. Spada F. Elsaesser J. Lahaye T. Leonhardt H. (2012). Targeted transcriptional activation of silent oct4 pluripotency gene by combining designer TALEs and inhibition of epigenetic modifiers. Nucleic Acids Res. 40, 5368–5377 22387464 10.1093/nar/gks199 PMC3384321

[b10-0060896] Carvajal-Vergara X. Sevilla A. D’Souza S. L. Ang Y. S. Schaniel C. Lee D. F. Yang L. Kaplan A. D. Adler E. D. Rozov R. (2010). Patient-specific induced pluripotent stem-cell-derived models of LEOPARD syndrome. Nature 465, 808–812 20535210 10.1038/nature09005 PMC2885001

[b11-0060896] Cermak T. Doyle E. L. Christian M. Wang L. Zhang Y. Schmidt C. Baller J. A. Somia N. V. Bogdanove A. J. Voytas D. F. (2011). Efficient design and assembly of custom TALEN and other TAL effector-based constructs for DNA targeting. Nucleic Acids Res. 39, e82 21493687 10.1093/nar/gkr218 PMC3130291

[b12-0060896] Cho S. W. Kim S. Kim J. M. Kim J. S. (2013). Targeted genome engineering in human cells with the Cas9 RNA-guided endonuclease. Nat. Biotechnol. 31, 230–232 23360966 10.1038/nbt.2507

[b13-0060896] Christian M. Cermak T. Doyle E. L. Schmidt C. Zhang F. Hummel A. Bogdanove A. J. Voytas D. F. (2010). Targeting DNA double-strand breaks with TAL effector nucleases. Genetics 186, 757–761 20660643 10.1534/genetics.110.120717 PMC2942870

[b14-0060896] Cong L. Ran F. A. Cox D. Lin S. Barretto R. Habib N. Hsu P. D. Wu X. Jiang W. Marraffini L. A. (2013). Multiplex genome engineering using CRISPR/Cas systems. Science 339, 819–823 23287718 10.1126/science.1231143 PMC3795411

[b15-0060896] Cowan C. A. Klimanskaya I. McMahon J. Atienza J. Witmyer J. Zucker J. P. Wang S. Morton C. C. McMahon A. P. Powers D. (2004). Derivation of embryonic stem-cell lines from human blastocysts. N. Engl. J. Med. 350, 1353–1356 14999088 10.1056/NEJMsr040330

[b16-0060896] Di Giorgio F. P. Boulting G. L. Bobrowicz S. Eggan K. C. (2008). Human embryonic stem cell-derived motor neurons are sensitive to the toxic effect of glial cells carrying an ALS-causing mutation. Cell Stem Cell 3, 637–648 19041780 10.1016/j.stem.2008.09.017

[b17-0060896] Dimos J. T. Rodolfa K. T. Niakan K. K. Weisenthal L. M. Mitsumoto H. Chung W. Croft G. F. Saphier G. Leibel R. Goland R. (2008). Induced pluripotent stem cells generated from patients with ALS can be differentiated into motor neurons. Science 321, 1218–1221 18669821 10.1126/science.1158799

[b18-0060896] Draper J. S. Smith K. Gokhale P. Moore H. D. Maltby E. Johnson J. Meisner L. Zwaka T. P. Thomson J. A. Andrews P. W. (2004). Recurrent gain of chromosomes 17q and 12 in cultured human embryonic stem cells. Nat. Biotechnol. 22, 53–54 14661028 10.1038/nbt922

[b19-0060896] Ebert A. D. Yu J. Rose F. F. Jr Mattis V. B. Lorson C. L. Thomson J. A. Svendsen C. N. (2009). Induced pluripotent stem cells from a spinal muscular atrophy patient. Nature 457, 277–280 19098894 10.1038/nature07677 PMC2659408

[b20-0060896] Gabriel R. Lombardo A. Arens A. Miller J. C. Genovese P. Kaeppel C. Nowrouzi A. Bartholomae C. C. Wang J. Friedman G. (2011). An unbiased genome-wide analysis of zinc-finger nuclease specificity. Nat. Biotechnol. 29, 816–823 21822255 10.1038/nbt.1948

[b21-0060896] Gore A. Li Z. Fung H. L. Young J. E. Agarwal S. Antosiewicz-Bourget J. Canto I. Giorgetti A. Israel M. A. Kiskinis E. (2011). Somatic coding mutations in human induced pluripotent stem cells. Nature 471, 63–67 21368825 10.1038/nature09805 PMC3074107

[b22-0060896] Grizot S. Smith J. Daboussi F. Prieto J. Redondo P. Merino N. Villate M. Thomas S. Lemaire L. Montoya G. (2009). Efficient targeting of a SCID gene by an engineered single-chain homing endonuclease. Nucleic Acids Res. 37, 5405–5419 19584299 10.1093/nar/gkp548 PMC2760784

[b23-0060896] Gupta A. Christensen R. G. Rayla A. L. Lakshmanan A. Stormo G. D. Wolfe S. A. (2012). An optimized two-finger archive for ZFN-mediated gene targeting. Nat. Methods 9, 588–590 22543349 10.1038/nmeth.1994 PMC3443678

[b24-0060896] HD iPSC Consortium (2012). Induced pluripotent stem cells from patients with Huntington’s disease show CAG-repeat-expansion-associated phenotypes. Cell Stem Cell 11, 264–278 22748968 10.1016/j.stem.2012.04.027 PMC3804072

[b25-0060896] Hockemeyer D. Soldner F. Beard C. Gao Q. Mitalipova M. DeKelver R. C. Katibah G. E. Amora R. Boydston E. A. Zeitler B. (2009). Efficient targeting of expressed and silent genes in human ESCs and iPSCs using zinc-finger nucleases. Nat. Biotechnol. 27, 851–857 19680244 10.1038/nbt.1562 PMC4142824

[b26-0060896] Hockemeyer D. Wang H. Kiani S. Lai C. S. Gao Q. Cassady J. P. Cost G. J. Zhang L. Santiago Y. Miller J. C. (2011). Genetic engineering of human pluripotent cells using TALE nucleases. Nat. Biotechnol. 29, 731–734 21738127 10.1038/nbt.1927 PMC3152587

[b27-0060896] Howden S. E. Gore A. Li Z. Fung H. L. Nisler B. S. Nie J. Chen G. McIntosh B. E. Gulbranson D. R. Diol N. R. (2011). Genetic correction and analysis of induced pluripotent stem cells from a patient with gyrate atrophy. Proc. Natl. Acad. Sci. USA 108, 6537–6542 21464322 10.1073/pnas.1103388108 PMC3080993

[b28-0060896] Hussein S. M. Batada N. N. Vuoristo S. Ching R. W. Autio R. Närvä E. Ng S. Sourour M. Hämäläinen R. Olsson C. (2011). Copy number variation and selection during reprogramming to pluripotency. Nature 471, 58–62 21368824 10.1038/nature09871

[b29-0060896] Ji J. Ng S. H. Sharma V. Neculai D. Hussein S. Sam M. Trinh Q. Church G. M. McPherson J. D. Nagy A. (2012). Elevated coding mutation rate during the reprogramming of human somatic cells into induced pluripotent stem cells. Stem Cells 30, 435–440 22162363 10.1002/stem.1011

[b30-0060896] Jinek M. East A. Cheng A. Lin S. Ma E. Doudna J. (2013). RNA-programmed genome editing in human cells. Elife 2, e00471 23386978 10.7554/eLife.00471 PMC3557905

[b31-0060896] Khan I. F. Hirata R. K. Wang P. R. Li Y. Kho J. Nelson A. Huo Y. Zavaljevski M. Ware C. Russell D. W. (2010). Engineering of human pluripotent stem cells by AAV-mediated gene targeting. Mol. Ther. 18, 1192–1199 20407427 10.1038/mt.2010.55 PMC2889726

[b32-0060896] Kim K. Doi A. Wen B. Ng K. Zhao R. Cahan P. Kim J. Aryee M. J. Ji H. Ehrlich L. I. (2010). Epigenetic memory in induced pluripotent stem cells. Nature 467, 285–290 20644535 10.1038/nature09342 PMC3150836

[b33-0060896] Kim K. Zhao R. Doi A. Ng K. Unternaehrer J. Cahan P. Huo H. Loh Y. H. Aryee M. J. Lensch M. W. (2011). Donor cell type can influence the epigenome and differentiation potential of human induced pluripotent stem cells. Nat. Biotechnol. 29, 1117–1119 22119740 10.1038/nbt.2052 PMC3357310

[b34-0060896] Laurent L. C. Ulitsky I. Slavin I. Tran H. Schork A. Morey R. Lynch C. Harness J. V. Lee S. Barrero M. J. (2011). Dynamic changes in the copy number of pluripotency and cell proliferation genes in human ESCs and iPSCs during reprogramming and time in culture. Cell Stem Cell 8, 106–118 21211785 10.1016/j.stem.2010.12.003 PMC3043464

[b35-0060896] Lee G. Papapetrou E. P. Kim H. Chambers S. M. Tomishima M. J. Fasano C. A. Ganat Y. M. Menon J. Shimizu F. Viale A. (2009). Modelling pathogenesis and treatment of familial dysautonomia using patient-specific iPSCs. Nature 461, 402–406 19693009 10.1038/nature08320 PMC2784695

[b36-0060896] Lee G. Chambers S. M. Tomishima M. J. Studer L. (2010). Derivation of neural crest cells from human pluripotent stem cells. Nat. Protoc. 5, 688–701 20360764 10.1038/nprot.2010.35

[b37-0060896] Li M. Suzuki K. Qu J. Saini P. Dubova I. Yi F. Lee J. Sancho-Martinez I. Liu G. H. Izpisua Belmonte J. C. (2011). Efficient correction of hemoglobinopathy-causing mutations by homologous recombination in integration-free patient iPSCs. Cell Res. 21, 1740–1744 22105484 10.1038/cr.2011.186 PMC3357996

[b38-0060896] Lian X. Zhang J. Azarin S. M. Zhu K. Hazeltine L. B. Bao X. Hsiao C. Kamp T. J. Palecek S. P. (2013). Directed cardiomyocyte differentiation from human pluripotent stem cells by modulating Wnt/β-catenin signaling under fully defined conditions. Nat. Protoc. 8, 162–175 23257984 10.1038/nprot.2012.150 PMC3612968

[b39-0060896] Lister R. Pelizzola M. Kida Y. S. Hawkins R. D. Nery J. R. Hon G. Antosiewicz-Bourget J. O’Malley R. Castanon R. Klugman S. (2011). Hotspots of aberrant epigenomic reprogramming in human induced pluripotent stem cells. Nature 471, 68–73 21289626 10.1038/nature09798 PMC3100360

[b40-0060896] Liu G. H. Suzuki K. Qu J. Sancho-Martinez I. Yi F. Li M. Kumar S. Nivet E. Kim J. Soligalla R. D. (2011). Targeted gene correction of laminopathy-associated LMNA mutations in patient-specific iPSCs. Cell Stem Cell 8, 688–694 21596650 10.1016/j.stem.2011.04.019 PMC3480729

[b41-0060896] Liu G. H. Qu J. Suzuki K. Nivet E. Li M. Montserrat N. Yi F. Xu X. Ruiz S. Zhang W. (2012). Progressive degeneration of human neural stem cells caused by pathogenic LRRK2. Nature 491, 603–607 23075850 10.1038/nature11557 PMC3504651

[b42-0060896] Loh Y. H. Hartung O. Li H. Guo C. Sahalie J. M. Manos P. D. Urbach A. Heffner G. C. Grskovic M. Vigneault F. (2010). Reprogramming of T cells from human peripheral blood. Cell Stem Cell 7, 15–19 20621044 10.1016/j.stem.2010.06.004 PMC2913590

[b43-0060896] Lombardo A. Genovese P. Beausejour C. M. Colleoni S. Lee Y. L. Kim K. A. Ando D. Urnov F. D. Galli C. Gregory P. D. (2007). Gene editing in human stem cells using zinc finger nucleases and integrase-defective lentiviral vector delivery. Nat. Biotechnol. 25, 1298–1306 17965707 10.1038/nbt1353

[b44-0060896] Maeder M. L. Thibodeau-Beganny S. Osiak A. Wright D. A. Anthony R. M. Eichtinger M. Jiang T. Foley J. E. Winfrey R. J. Townsend J. A. (2008). Rapid ‘open-source’ engineering of customized zinc-finger nucleases for highly efficient gene modification. Mol. Cell 31, 294–301 18657511 10.1016/j.molcel.2008.06.016 PMC2535758

[b45-0060896] Maeder M. L. Thibodeau-Beganny S. Sander J. D. Voytas D. F. Joung J. K. (2009). Oligomerized pool engineering (OPEN): an ‘open-source’ protocol for making customized zinc-finger arrays. Nat. Protoc. 4, 1471–1501 19798082 10.1038/nprot.2009.98 PMC2858690

[b46-0060896] Maitra A. Arking D. E. Shivapurkar N. Ikeda M. Stastny V. Kassauei K. Sui G. Cutler D. J. Liu Y. Brimble S. N. (2005). Genomic alterations in cultured human embryonic stem cells. Nat. Genet. 37, 1099–1103 16142235 10.1038/ng1631

[b47-0060896] Mali P. Yang L. Esvelt K. M. Aach J. Guell M. DiCarlo J. E. Norville J. E. Church G. M. (2013). RNA-guided human genome engineering via Cas9. Science 339, 823–826 23287722 10.1126/science.1232033 PMC3712628

[b48-0060896] Marchetto M. C. Carromeu C. Acab A. Yu D. Yeo G. W. Mu Y. Chen G. Gage F. H. Muotri A. R. (2010). A model for neural development and treatment of Rett syndrome using human induced pluripotent stem cells. Cell 143, 527–539 21074045 10.1016/j.cell.2010.10.016 PMC3003590

[b49-0060896] Martins-Taylor K. Nisler B. S. Taapken S. M. Compton T. Crandall L. Montgomery K. D. Lalande M. Xu R. H. (2011). Recurrent copy number variations in human induced pluripotent stem cells. Nat. Biotechnol. 29, 488–491 21654665 10.1038/nbt.1890

[b50-0060896] Mayshar Y. Ben-David U. Lavon N. Biancotti J. C. Yakir B. Clark A. T. Plath K. Lowry W. E. Benvenisty N. (2010). Identification and classification of chromosomal aberrations in human induced pluripotent stem cells. Cell Stem Cell 7, 521–531 20887957 10.1016/j.stem.2010.07.017

[b51-0060896] Miller J. C. Tan S. Qiao G. Barlow K. A. Wang J. Xia D. F. Meng X. Paschon D. E. Leung E. Hinkley S. J. (2011). A TALE nuclease architecture for efficient genome editing. Nat. Biotechnol. 29, 143–148 21179091 10.1038/nbt.1755

[b52-0060896] Mitalipova M. M. Rao R. R. Hoyer D. M. Johnson J. A. Meisner L. F. Jones K. L. Dalton S. Stice S. L. (2005). Preserving the genetic integrity of human embryonic stem cells. Nat. Biotechnol. 23, 19–20 15637610 10.1038/nbt0105-19

[b53-0060896] Moretti A. Bellin M. Welling A. Jung C. B. Lam J. T. Bott-Flügel L. Dorn T. Goedel A. Höhnke C. Hofmann F. (2010). Patient-specific induced pluripotent stem-cell models for long-QT syndrome. N. Engl. J. Med. 363, 1397–1409 20660394 10.1056/NEJMoa0908679

[b54-0060896] Moscou M. J. Bogdanove A. J. (2009). A simple cipher governs DNA recognition by TAL effectors. Science 326, 1501 19933106 10.1126/science.1178817

[b55-0060896] Nakagawa M. Koyanagi M. Tanabe K. Takahashi K. Ichisaka T. Aoi T. Okita K. Mochiduki Y. Takizawa N. Yamanaka S. (2008). Generation of induced pluripotent stem cells without Myc from mouse and human fibroblasts. Nat. Biotechnol. 26, 101–106 18059259 10.1038/nbt1374

[b56-0060896] Nazor K. L. Altun G. Lynch C. Tran H. Harness J. V. Slavin I. Garitaonandia I. Müller F. J. Wang Y. C. Boscolo F. S. (2012). Recurrent variations in DNA methylation in human pluripotent stem cells and their differentiated derivatives. Cell Stem Cell 10, 620–634 22560082 10.1016/j.stem.2012.02.013 PMC3348513

[b57-0060896] Nishino K. Toyoda M. Yamazaki-Inoue M. Fukawatase Y. Chikazawa E. Sakaguchi H. Akutsu H. Umezawa A. (2011). DNA methylation dynamics in human induced pluripotent stem cells over time. PLoS Genet. 7, e1002085 21637780 10.1371/journal.pgen.1002085 PMC3102737

[b58-0060896] Park I. H. Zhao R. West J. A. Yabuuchi A. Huo H. Ince T. A. Lerou P. H. Lensch M. W. Daley G. Q. (2008a). Reprogramming of human somatic cells to pluripotency with defined factors. Nature 451, 141–146 18157115 10.1038/nature06534

[b59-0060896] Park I. H. Arora N. Huo H. Maherali N. Ahfeldt T. Shimamura A. Lensch M. W. Cowan C. Hochedlinger K. Daley G. Q. (2008b). Disease-specific induced pluripotent stem cells. Cell 134, 877–886 18691744 10.1016/j.cell.2008.07.041 PMC2633781

[b60-0060896] Pattanayak V. Ramirez C. L. Joung J. K. Liu D. R. (2011). Revealing off-target cleavage specificities of zinc-finger nucleases by in vitro selection. Nat. Methods 8, 765–770 21822273 10.1038/nmeth.1670 PMC3164905

[b61-0060896] Perez E. E. Wang J. Miller J. C. Jouvenot Y. Kim K. A. Liu O. Wang N. Lee G. Bartsevich V. V. Lee Y. L. (2008). Establishment of HIV-1 resistance in CD4+ T cells by genome editing using zinc-finger nucleases. Nat. Biotechnol. 26, 808–816 18587387 10.1038/nbt1410 PMC3422503

[b62-0060896] Polo J. M. Liu S. Figueroa M. E. Kulalert W. Eminli S. Tan K. Y. Apostolou E. Stadtfeld M. Li Y. Shioda T. (2010). Cell type of origin influences the molecular and functional properties of mouse induced pluripotent stem cells. Nat. Biotechnol. 28, 848–855 20644536 10.1038/nbt.1667 PMC3148605

[b63-0060896] Qiu S. Adema C. M. Lane T. (2005). A computational study of off-target effects of RNA interference. Nucleic Acids Res. 33, 1834–1847 15800213 10.1093/nar/gki324 PMC1072799

[b64-0060896] Ramirez C. L. Foley J. E. Wright D. A. Müller-Lerch F. Rahman S. H. Cornu T. I. Winfrey R. J. Sander J. D. Fu F. Townsend J. A. (2008). Unexpected failure rates for modular assembly of engineered zinc fingers. Nat. Methods 5, 374–375 18446154 10.1038/nmeth0508-374 PMC7880305

[b65-0060896] Rashid S. T. Corbineau S. Hannan N. Marciniak S. J. Miranda E. Alexander G. Huang-Doran I. Griffin J. Ahrlund-Richter L. Skepper J. (2010). Modeling inherited metabolic disorders of the liver using human induced pluripotent stem cells. J. Clin. Invest. 120, 3127–3136 20739751 10.1172/JCI43122 PMC2929734

[b66-0060896] Reinhardt P. Schmid B. Burbulla L. F. Schöndorf D. C. Wagner L. Glatza M. Höing S. Hargus G. Heck S. A. Dhingra A. (2013). Genetic correction of a LRRK2 mutation in human iPSCs links parkinsonian neurodegeneration to ERK-dependent changes in gene expression. Cell Stem Cell 12, 354–367 23472874 10.1016/j.stem.2013.01.008

[b67-0060896] Reubinoff B. E. Pera M. F. Fong C. Y. Trounson A. Bongso A. (2000). Embryonic stem cell lines from human blastocysts: somatic differentiation in vitro. Nat. Biotechnol. 18, 399–404 10748519 10.1038/74447

[b68-0060896] Reyon D. Tsai S. Q. Khayter C. Foden J. A. Sander J. D. Joung J. K. (2012). FLASH assembly of TALENs for high-throughput genome editing. Nat. Biotechnol. 30, 460–465 22484455 10.1038/nbt.2170 PMC3558947

[b69-0060896] Ruiz S. Diep D. Gore A. Panopoulos A. D. Montserrat N. Plongthongkum N. Kumar S. Fung H. L. Giorgetti A. Bilic J. (2012). Identification of a specific reprogramming-associated epigenetic signature in human induced pluripotent stem cells. Proc. Natl. Acad. Sci. USA 109, 16196–16201 22991473 10.1073/pnas.1202352109 PMC3479609

[b70-0060896] Russell D. W. Hirata R. K. (1998). Human gene targeting by viral vectors. Nat. Genet. 18, 325–330 9537413 10.1038/ng0498-325 PMC3010411

[b71-0060896] Sander J. D. Dahlborg E. J. Goodwin M. J. Cade L. Zhang F. Cifuentes D. Curtin S. J. Blackburn J. S. Thibodeau-Beganny S. Qi Y. (2011). Selection-free zinc-finger-nuclease engineering by context-dependent assembly (CoDA). Nat. Methods 8, 67–69 21151135 10.1038/nmeth.1542 PMC3018472

[b72-0060896] Sanjana N. E. Cong L. Zhou Y. Cunniff M. M. Feng G. Zhang F. (2012). A transcription activator-like effector toolbox for genome engineering. Nat. Protoc. 7, 171–192 22222791 10.1038/nprot.2011.431 PMC3684555

[b73-0060896] Sebastiano V. Maeder M. L. Angstman J. F. Haddad B. Khayter C. Yeo D. T. Goodwin M. J. Hawkins J. S. Ramirez C. L. Batista L. F. (2011). In situ genetic correction of the sickle cell anemia mutation in human induced pluripotent stem cells using engineered zinc finger nucleases. Stem Cells 29, 1717–1726 21898685 10.1002/stem.718 PMC3285772

[b74-0060896] Seki T. Yuasa S. Oda M. Egashira T. Yae K. Kusumoto D. Nakata H. Tohyama S. Hashimoto H. Kodaira M. (2010). Generation of induced pluripotent stem cells from human terminally differentiated circulating T cells. Cell Stem Cell 7, 11–14 20621043 10.1016/j.stem.2010.06.003

[b75-0060896] Smithies O. Gregg R. G. Boggs S. S. Koralewski M. A. Kucherlapati R. S. (1985). Insertion of DNA sequences into the human chromosomal β-globin locus by homologous recombination. Nature 317, 230–234 2995814 10.1038/317230a0

[b76-0060896] Soldner F. Laganière J. Cheng A. W. Hockemeyer D. Gao Q. Alagappan R. Khurana V. Golbe L. I. Myers R. H. Lindquist S. (2011). Generation of isogenic pluripotent stem cells differing exclusively at two early onset Parkinson point mutations. Cell 146, 318–331 21757228 10.1016/j.cell.2011.06.019 PMC3155290

[b77-0060896] Staerk J. Dawlaty M. M. Gao Q. Maetzel D. Hanna J. Sommer C. A. Mostoslavsky G. Jaenisch R. (2010). Reprogramming of human peripheral blood cells to induced pluripotent stem cells. Cell Stem Cell 7, 20–24 20621045 10.1016/j.stem.2010.06.002 PMC2917234

[b78-0060896] Sun N. Yazawa M. Liu J. Han L. Sanchez-Freire V. Abilez O. J. Navarrete E. G. Hu S. Wang L. Lee A. (2012). Patient-specific induced pluripotent stem cells as a model for familial dilated cardiomyopathy. Sci. Transl. Med. 4, 130ra47 10.1126/scitranslmed.3003552 PMC3657516 22517884

[b79-0060896] Suzuki K. Mitsui K. Aizawa E. Hasegawa K. Kawase E. Yamagishi T. Shimizu Y. Suemori H. Nakatsuji N. Mitani K. (2008). Highly efficient transient gene expression and gene targeting in primate embryonic stem cells with helper-dependent adenoviral vectors. Proc. Natl. Acad. Sci. USA 105, 13781–13786 18768795 10.1073/pnas.0806976105 PMC2544531

[b80-0060896] Swistowski A. Peng J. Liu Q. Mali P. Rao M. S. Cheng L. Zeng X. (2010). Efficient generation of functional dopaminergic neurons from human induced pluripotent stem cells under defined conditions. Stem Cells 28, 1893–1904 20715183 10.1002/stem.499 PMC2996088

[b81-0060896] Taapken S. M. Nisler B. S. Newton M. A. Sampsell-Barron T. L. Leonhard K. A. McIntire E. M. Montgomery K. D. (2011). Karotypic abnormalities in human induced pluripotent stem cells and embryonic stem cells. Nat. Biotechnol. 29, 313–314 21478842 10.1038/nbt.1835

[b82-0060896] Tachibana M. Amato P. Sparman M. Gutierrez N. M. Tippner-Hedges R. Ma H. Kang E. Fulati A. Lee H. S. Sritanaudomchai H. (2013). Human embryonic stem cells derived by somatic cell nuclear transfer. Cell. 10.1016/j.cell.2013.05.006 PMC3772789 23683578

[b83-0060896] Takahashi K. Tanabe K. Ohnuki M. Narita M. Ichisaka T. Tomoda K. Yamanaka S. (2007). Induction of pluripotent stem cells from adult human fibroblasts by defined factors. Cell 131, 861–872 18035408 10.1016/j.cell.2007.11.019

[b84-0060896] Thomas K. R. Capecchi M. R. (1987). Site-directed mutagenesis by gene targeting in mouse embryo-derived stem cells. Cell 51, 503–512 2822260 10.1016/0092-8674(87)90646-5

[b85-0060896] Thomson J. A. Itskovitz-Eldor J. Shapiro S. S. Waknitz M. A. Swiergiel J. J. Marshall V. S. Jones J. M. (1998). Embryonic stem cell lines derived from human blastocysts. Science 282, 1145–1147 9804556 10.1126/science.282.5391.1145

[b86-0060896] Urnov F. D. Rebar E. J. Holmes M. C. Zhang H. S. Gregory P. D. (2010). Genome editing with engineered zinc finger nucleases. Nat. Rev. Genet. 11, 636–646 20717154 10.1038/nrg2842

[b87-0060896] Ye Z. Zhan H. Mali P. Dowey S. Williams D. M. Jang Y. Y. Dang C. V. Spivak J. L. Moliterno A. R. Cheng L. (2009). Human-induced pluripotent stem cells from blood cells of healthy donors and patients with acquired blood disorders. Blood 114, 5473–5480 19797525 10.1182/blood-2009-04-217406 PMC2798863

[b88-0060896] Yu J. Vodyanik M. A. Smuga-Otto K. Antosiewicz-Bourget J. Frane J. L. Tian S. Nie J. Jonsdottir G. A. Ruotti V. Stewart R. (2007). Induced pluripotent stem cell lines derived from human somatic cells. Science 318, 1917–1920 18029452 10.1126/science.1151526

[b89-0060896] Yusa K. Rashid S. T. Strick-Marchand H. Varela I. Liu P. Q. Paschon D. E. Miranda E. Ordóñez A. Hannan N. R. Rouhani F. J. (2011). Targeted gene correction of α1-antitrypsin deficiency in induced pluripotent stem cells. Nature 478, 391–394 21993621 10.1038/nature10424 PMC3198846

[b90-0060896] Zhang F. Cong L. Lodato S. Kosuri S. Church G. M. Arlotta P. (2011). Efficient construction of sequence-specific TAL effectors for modulating mammalian transcription. Nat. Biotechnol. 29, 149–153 21248753 10.1038/nbt.1775 PMC3084533

[b91-0060896] Zou J. Maeder M. L. Mali P. Pruett-Miller S. M. Thibodeau-Beganny S. Chou B. K. Chen G. Ye Z. Park I. H. Daley G. Q. (2009). Gene targeting of a disease-related gene in human induced pluripotent stem and embryonic stem cells. Cell Stem Cell 5, 97–110 19540188 10.1016/j.stem.2009.05.023 PMC2720132

[b92-0060896] Zou J. Mali P. Huang X. Dowey S. N. Cheng L. (2011). Site-specific gene correction of a point mutation in human iPS cells derived from an adult patient with sickle cell disease. Blood 118, 4599–4608 21881051 10.1182/blood-2011-02-335554 PMC3208277

[b93-0060896] Zwaka T. P. Thomson J. A. (2003). Homologous recombination in human embryonic stem cells. Nat. Biotechnol. 21, 319–321 12577066 10.1038/nbt788

